# 58896 Feasibility of a Parent Navigator Program for Parents of Justice-Involved Youth

**DOI:** 10.1017/cts.2021.545

**Published:** 2021-03-30

**Authors:** Allyson L. Dir, Sarah Wiehe, Matthew C. Aalsma

**Affiliations:** Indiana University School of Medicine

## Abstract

ABSTRACT IMPACT: Development and implementation of a parent navigator program to help parents of justice-involved youth could assist parents in navigating the justice system, improve engagement with court and probation, and ultimately improve outcomes for youth involved in the juvenile justice system OBJECTIVES/GOALS: The goals of the study are to (1) develop a parent-peer navigator program utilizing community-based participatory design; and (2) implement and assess the feasibility of a parent peer navigator program in an urban juvenile justice system. METHODS/STUDY POPULATION: The EPIS framework will guide development and implementation of the navigator program as well as measurement of the implementation process, including measurements of feasibility and acceptability. In the Exploration phase, qualitative interviews with juvenile justice staff, parents of justice-involved youth, and members of the local family advisory board will inform program needs. In the preparation stage, I will work closely with the family advisory board to develop the actual parent navigator program protocol, including a training plan for navigators and their specific roles. I will conduct an open trial in the implementation phase, measuring program feasibility and acceptability among parents, navigators, juvenile justice staff, parents, and youth utilizing mixed methods. RESULTS/ANTICIPATED RESULTS: Results will inform feasibility of implementing the program as well as acceptability of the program based on mixed methods data from parents of justice-involved youth, juvenile justice staff, family advisory board members, and other community stakeholders. Results will potentially inform conduct of a larger scale pilot hybrid implementation-effectiveness study. DISCUSSION/SIGNIFICANCE OF FINDINGS: Development and implementation of a parent navigator program to help parents of justice-involved youth could assist parents in navigating the justice system, improve engagement with court and probation, and ultimately improve outcomes for youth involved in the juvenile justice system.

